# Do Physical Activity, BMI, and Wellbeing Affect Logical Thinking?

**DOI:** 10.3390/ijerph19116631

**Published:** 2022-05-29

**Authors:** Albertas Skurvydas, Ausra Lisinskiene, Daiva Majauskiene, Dovile Valanciene, Ruta Dadeliene, Natalja Fatkulina, Asta Sarkauskiene

**Affiliations:** 1 Institute of Educational Research, Education Academy, Vytautas Magnus University, K. Donelaičio Street 58, 44248 Kaunas, Lithuania; a.skurvydas60@gmail.com (A.S.); ausra.lisinskiene@vdu.lt (A.L.); daiva.majauskiene@vdu.lt (D.M.); 2 Department of Rehabilitation, Physical and Sports Medicine, Faculty of Medicine, Institute of Health Sciences, Vilnius University, 21/27 M.K. Čiurlionio St., 03101 Vilnius, Lithuania; ruta.dadeliene@mf.vu.lt; 3 Institute of Health Sciences, Faculty of Medicine, Vilnius University, 21/27 M.K. Čiurlionio Street, 03101 Vilnius, Lithuania; natalja.fatkulina@mf.vu.lt; 4 Department of Sports, Recreation and Tourism, Klaipėda University, Herkaus Manto Street 84, 92294 Klaipėda, Lithuania; asta.sarkauskiene@ku.lt

**Keywords:** logical thinking, emotional intelligence, healthy lifestyle, mental health, physical activity, professional athletes

## Abstract

We studied 6368 people (4544 women and 1824 men; aged 18–74 years). The research goal was to determine whether the Cognitive Reflection Test score (logical thinking compared with intuitive thinking) depends—and in what way it depends—on the healthy lifestyle components and emotional health-related components as well as age (18–74 years) and gender. We established that analytical vs. intuitive thinking depended on components of a healthy lifestyle, physical activity, sleep, eating habits, smoking and alcohol consumption, specificity of sporting activity, body mass index, and emotional health-related components (stress, depression, impulsivity, subjective health, emotional intelligence), as well as age and gender. We found that logical thinking was not associated with sleep, moderate-to-vigorous PA, impulsivity, subjective health, and components of a healthy lifestyle. However, logical thinking decreases with age, gender (higher in men than in women), BMI (decreases in both genders over the second degree of obesity), depression (the more severe depression in women, the worse their logical thinking), sedentary behavior (people who sat for longer periods had more difficulty solving problems), and in professional sportswomen (logical thinking is worse in professional sportswomen than in sedentary women, amateur sportswomen, or women who use gyms). Finally, we determined inverse correlations between logical thinking, emotional intelligence, and stress.

## 1. Introduction

There is growing evidence that various forms and intensities of physical activity (PA) are effective in combating many chronic diseases [1,2] and improving wellbeing and mental health [3,4,5,6,7,8,9]. The health benefits of PA depend on age, gender, health status, and body mass index (BMI) [2,6,10,11]. Obesity increases due to inadequate PA and later causes systemic inflammation, leading to many chronic diseases [1,12]. Guthold et al. (2018) summarized the dynamics of physical inactivity for 1.9 million people from 2001 to 2016 and found that physical inactivity increased significantly in both men and women in developed European countries [13]. Obesity and low PA may be associated. For example, low PA promotes obesity, which in turn reduces motivation to perform PA [14] and encourages more frequent overeating because people cannot control their appetite [15]. Studies have shown that even during the COVID-19 pandemic, moderate PA (MPA), vigorous PA (VPA), or a combination of MPA and VPA (MVPA) did not change significantly, men and women reported eating less excessively than before, did not start consuming more alcohol, and their body mass index did not change [16].

Studies have shown that cognition can be affected by the following factors: hypertension, dyslipidemia, midlife obesity, diabetes mellitus, smoking, physical inactivity, depression, and low educational level [17,18]. Thus, executive functions such as decision-making, cognitive flexibility, and behavioral control are critical for adaptive success in all areas of life, including maintaining a healthy body weight [19]. PA also has well-documented benefits for cognitive health across the lifespan, including efficacy in maintaining cognitive function and partially reversing deterioration in cognitive function with disease or aging [20,21]. However, there is no consensus on the effect of PA on cognition. For example, our study and the studies by Brown et al. (2021) have not shown that cognitive abilities of older people are improved by PA [22,23]. There is often an inverse relationship between BMI, mental health, and cognition: for example, obesity and low BMI can worsen mental health [24,25,26,27,28,29,30]. There is a direct relationship between skeletal muscle health and cognition [31]. For example, aging plays a role in both skeletal muscle decline and cognitive decline. In addition, obesity accelerates the decline in cognitive function with age [32,33]. However, the use of PA can counteract this decline [18,34]. A meta-analysis showed that higher levels of PA reduce the risk of cognitive decline and dementia [35]. There is also an inverse correlation between the extent of manifestation of chronic diseases and people’s intelligence quotient (IQ) [36]. Thus, higher IQ in adolescence was associated with healthier behavioral patterns in middle age [37]. However, intelligence in early adulthood was found to be inversely associated with the level of PA [38]. Many authors have emphasized the distinction between two types of cognitive processes: those that are executed rapidly (i.e., “fast” thinking) with little conscious deliberation and those that are slower and more reflective (i.e., “slow” reflective thinking) [39,40]. Dual-process models assume that health actions are controlled not only by a conscious, reflective, rule-based system, but also by an unconscious, impulsive, associative system [41]. Explicit (“slow” reflective) thinking requires more effort than implicit (“fast”) thinking; therefore, implicit thinking is used more often when making fast decisions [40,42]. Multimodal fitness and cognitive training improves fluid intelligence, although fitness training alone with PA cannot improve intelligence [43].

Thus, there is evidence that PA, sedentary behavior (SB) in terms of sitting time, and obesity may impact cognition (executive function), but there is no research on how they affect intuitive thinking versus LT and in what way LT, compared with intuitive thinking, depends on the components of healthy lifestyle forms of PA (sleeping and eating habits, smoking and alcohol consumption, specificity of sporting activity, and BMI), emotional health-related components (stress, depression, impulsivity, subjective health, and EI), as well as age and gender. We used the cognitive reflection test (CRT) to determine the effectiveness of reflective thinking [44]. The CRT is applied widely in cognitive ability research (intuitive thinking and LT) [45,46,47,48,49]. Therefore, our research goal was to determine whether the CRT score (LT compared with intuitive thinking) depends—and in what way it depends—on the above-mentioned healthy lifestyle components and emotional health-related components, as well as age (18–74 years) and gender.

## 2. Materials and Methods

### 2.1. Participants

Participants were 6368 research participants (females = 4544 and males = 1824) between the ages of 18 and 74 years old, correctly completed. The research was conducted from October 2019 to June 2020. Participants were from the country of Lithuania to represent the Lithuanian sample. Participants were randomly selected for the study and voluntarily agreed to participate in the study by completing the questionnaire. Participation was anonymous and included a brief description of the reasons for participating in the study, so data collection and processing were confidential. We used an online survey to collect information (https://docs.google.com/forms/ accessed on 1 October 2019). All participants completed the online questionnaires. An online survey using the Google Forms platform was distributed by researchers through social media (Facebook) and personal contacts (WhatsApp). Using the survey, we determined the BMI and specificity of PA of the participants.

### 2.2. Procedure

The Ethics Committee of the University of Klaipeda approved the conduct of this study (Protocol No. STIMC-BTMEK-08). We also ensured that the study was conducted according to the principles of the Declaration of Helsinki [50] and the National Guidelines for Biomedical and Health Research with Human Participants [51]. The purpose of the survey, the introduction, and the length of the survey were added to the web-based open e-survey. Successful return of the completed survey was considered participant consent.

### 2.3. Measurements

*Danish Physical Activity Questionnaire (DPAQ).* We used a quantitative, cross-sectional study design. The following instruments were used in the conduct of this study: the DPAQ was adapted from the International Physical Activity Questionnaire (IPAQ; https://loinc.org/77582-5/ (accessed on 25 August 2019)) and differs from it in that it refers to the PA of the last 24 h for 7 consecutive days, rather than simply the last 7 days [52]. The selected activities are listed in the PA scale in nine levels of physical effort, ranging from sleep or SB (0.9 MET) to strenuous activities (>6 METs). Each level in terms of task metabolic activity values (MET) (A = 0.9, B = 1.0, C = 1.5, D = 2.0, E = 3.0, F = 4.0, G = 5.0, H = 6.0, and I > 6) is described in the DPAQ by examples of specific activities for that level and by a small drawing. The PA scale was constructed to indicate the number of minutes (15, 30, or 45) and hours (1–10) spent at each MET activity level in an average 24 h weekday. This allowed calculation of the total MET time representing 24 h of sleep, work, and leisure on an average weekday [53].

We calculated how much energy was expended in the form of METs per day during sleep, SB (0.9–1.5 METs), light-intensity PA (LPA; >1.5 <3 METs), moderate-intensity PA (MPA; 3 to <6 METs), and vigorous-intensity PA (VPA; >6 METs). We also combined MPA and VPA as MVPA and calculated how many METs were wasted when intensity was >6 METs: as extra-vigorous PA (VPAextra).

*The Cognitive Reflection Test (CRT).* The test items were developed following the CRT test discussed in the article by Frederick (2005) [44]. The test consists of three tasks in which the wrong answer is automatically selected after reading. The author states that it is possible to check what kind of thinking system a person uses. The first system reflects intuitive decision-making, which is usually fast, automatic, requires minimal effort, is implicit, and is emotional. The second system, on the other hand, reflects thinking that is slower, more deliberate, requires more effort, is goal-oriented, and is more logical. The test consists of three questions, for example: (1) A bat and a ball together cost $1.10. The bat costs $1.00 more than the ball. How much does the ball cost? _____ cents; (2) If it takes 5 machines 5 min to make 5 widgets, how long would it take 100 machines to make 100 widgets? _____ minutes; (3) In a lake, there is a patch of lily pads in a lake. Every day the patch doubles in size. If it takes 48 days for the patch to cover the entire lake, how long would it take for the patch to cover half of the lake? _____ days. The measure is scored as the total number of correct answers. The cognitive reflection test (CRT) measures the cognitive process, i.e., the tendency to suppress an incorrect, intuitive response and arrive at a more conscious, correct response.

*Assessment of emotional intelligence*. Emotional intelligence was assessed using the Schutte Self-Report Emotional Intelligence Test (SSREIT) [54]. The SSREIT is a 33-item questionnaire divided into four subscales: perception of emotions (10 items), dealing with one’s own emotions (9 items), dealing with others’ emotions (8 items), and using emotions (5 items). The items are answered on a five-point scale ranging from 1 (strongly agree) to 5 (strongly agree). Total scores range from 33 to 165, with higher scores indicating greater ability in the area of EI.

*Perceived stress and depression*. The 10-item Perceived Stress Scale (PSS-10) was used to measure participants’ stress levels [55]. In the PSS-10, participants were asked to answer 10 questions about their feelings and thoughts in the past month on a Likert scale ranging from 0 (never) to 4 (very often) to indicate how often they felt or have felt a certain way in the past month. Scores range from 0 to 4, with higher scores indicating higher levels of perceived stress.

*Subjective depression self-assessment.* Each item was assessed on a four point (0–3) response category: was not overwhelmed by depression (0 point); depression was more prevalent than before (1 points); depression was prevalent slightly more frequently than before (2 points); depression covered much more often than before (3 points).

*Subjective health assessment*. A four-point scale was used for this: poor health (1 point); satisfactory (2 points); good (3 points); excellent (4 points).

*Assessment of impulsivity*. Impulsivity was assessed using the Barratt Impulsivity Scale version 11 (BIS-11) [56]. The BIS-11 is a 30-item questionnaire divided into three subscales: attentional impulsivity, scored with 8 items; motor impulsivity, scored with 11 items; and non-planning impulsivity, scored with 11 items. Items are answered on a four-point scale ranging from 1 (rarely/never) to 4 (almost always/always). Total scores range from 30 to 120, with higher scores representing higher impulsivity.

### 2.4. Statistical Analysis

Interval data were expressed as mean ± standard error. All data were confirmed as normally distributed using the Kolmogorov–Smirnov test. Three-way analyses of variance (ANOVAs) were performed to assess the effects of the independent variables (CRT score, age, gender) on the dependent variables (BMI, EI, SB, MPA, VPA). Three-way ANOVA was performed to evaluate the effect of the independent variables (specificity of exercise type, age, gender) on the dependent variable (CRT score). The observed power (OP) was also calculated. The value of the partial eta squared (ŋ_P^2) was estimated as a measure of effect size. When significant effects were found, Tukey post hoc adjustment was used for multiple comparisons within each measurement replicate ANOVA. We also calculated Pearson’s correlation coefficient. For all tests, statistical significance was defined as *p* < 0.05. Statistical analyses were performed using IBM SPSS Statistics software (v. 22; IBM Corp., Armonk, NY, USA).

For the effect of age on the results of CRT, we divided age into the following categories: <25, 25–34.9, 35–44.9, 45–55, and >55 years. For the relationships between BMI and the CRT test, we divided BMI into the following categories: <18, 18–24.9, 25–29.9, 30–35, and >35 kg/m^2^. For the MVPA and CRT score, we divided MVPA into the following categories: 0, >0–10, >10–20, and >10 metabolic equivalents of task per hour (METs-h) per day. We also determined relationships between the results of CRT and educational level, residential location and house, marital status, type of work, overeating, eating breakfast, health level, MVPA, smoking, alcohol consumption, stress level, impulsivity, depression, and sleep. In all cases, we calculated chi-squared (χ^2^) and *p* values for men and women separately.

## 3. Results

### 3.1. Effect of Age and Gender on CRT Scores

**Figure 1:**
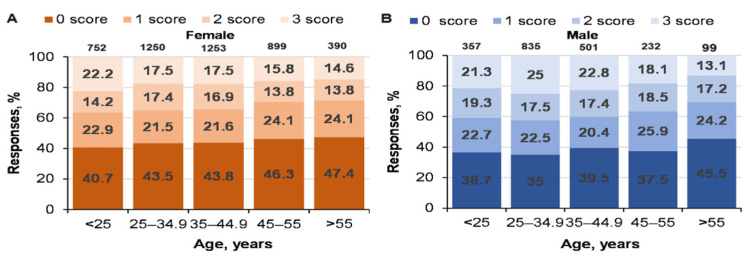
Dynamics of age-related performance in solving CRT tasks for men and women. At the top of the column is the number of participants. ((**A**)—women CRT responses % and age; (**B**)—men CRT responses % and age).

The influence of age on solving CRT tasks was only significant for women (χ^2^ = 26.2; *p* = 0.009 vs. male subjects, χ^2^ = 14.2; *p* = 0.283; Figure 1). Eighteen- to 25-year-old women (the youngest age group) and the 25- to 35-year-old men solved the CRT tasks better than the other age groups. The solution for these three tasks decreased in men and women with age, but the numbers of men and women who did not solve any tasks increased significantly (*p* < 0.05). The CRT score in women was lower than in men for all age groups (*p* < 0.05) (Figure 1).

### 3.2. Relationship between BMI and CRT Results

**Figure 2:**
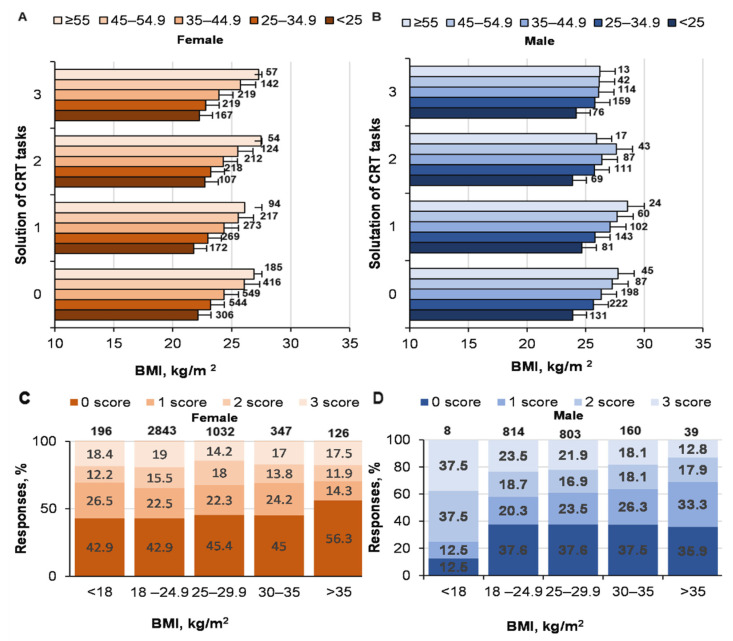
Solving CRT tasks and BMI. At the top of the column is the number of participants. ((**A**)—women solutions of CRT and BMI; (**B**)—men solutions of CRT and BMI; (**C**)—women CRT responses % and BMI; (**D**)—men CRT responses % and BMI).

There were no statistically significant relationships between the effectiveness of solving CRT tasks and BMI (effect of logic on BMI: *p* = 0.278; ${ŋ}_{P}^{2}\;$< 0.001; OP = 0.346), but BMI depended on age (*p* < 0.001; ${ŋ}_{P}^{2}$ = 0.053; OP = 1) and gender (*p* < 0.001; ${ŋ}_{P}^{2}$ = 0.021; OP = 1) (Figure 2). The interaction effect of logic with gender was as significant (*p* = 0.003; $\;{ŋ}_{P}^{2}$ = 0.002; OP = 0.94) as the interaction of logic with age (*p* < 0.001; $\;{ŋ}_{P}^{2}$ = 0.004; OP = 1).

The influence of BMI on solving CRT tasks was only significant for women (female, χ^2^ = 28.1; *p* = 0.005; male, χ^2^ = 13.5; *p* = 0.33). The failure to complete at least one task especially increased in women with BMI > 35 kg/m^2^ (*p* < 0.05 compared with women in other BMI categories), whereas the solutions for all tasks decreased significantly among men with the same BMI category (*p* < 0.05). The number of men in this BMI category who solved three tasks decreased, but the number of those that solved one task increased. Interestingly, men with the lowest BMI correctly solved the maximum number of three CRT tasks compared with the other groups. As the BMI of the men increased, the number of those who correctly solved three tasks decreased. There was no significant correlation between the effectiveness of solving CRT tasks and BMI (r = −0.018; *p* > 0.05).

### 3.3. Relationship between PA and CRT Results

**Figure 3:**
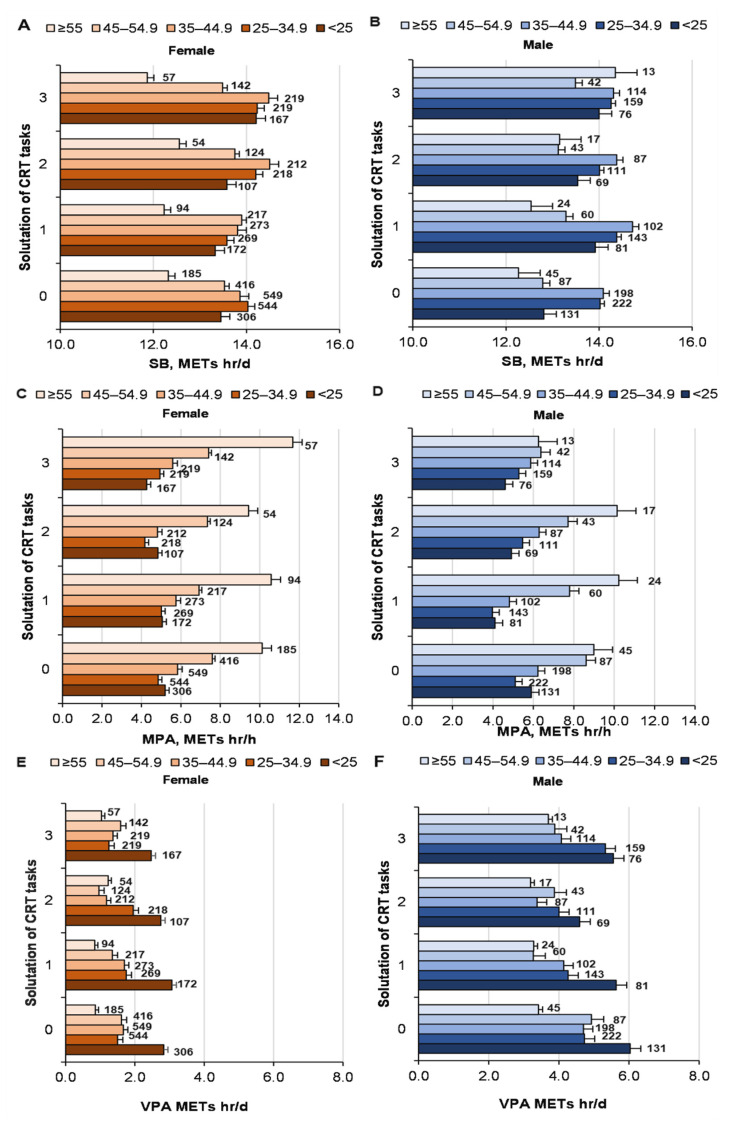
Solving CRT problems by men and women in different age groups with PA (SB, MPA, VPA, MVPA). At the top of the column is the number of participants. ((**A**)—women solutions of CRT and SB; (**B**)—men solutions of CRT and SB; (**C**)—women solutions of CRT and MPA; (**D**)—men solutions of CRT and MPA; (**E**)—women solutions of CRT and VPA; (**F**)—men solutions of CRT and VPA).

There was a tendency for a direct relationship between solving CRT tasks and SB (*p* = 0.016; ${ŋ}_{P}^{2}$ = 0.002; OP = 0.76) (Figure 3). SB depended on age (*p* < 0.001; ${ŋ}_{P}^{2}$ = 0.009; OP = 1), but not on gender (*p* = 0.36; $\;{ŋ}_{P}^{2}$ < 0.001; OP = 0.15). We determined a statistically significant correlation (Pearson coefficient) between the effectiveness of solving CRT tasks and SB (r = −0.045; *p* < 0.01).

**Figure 4:**
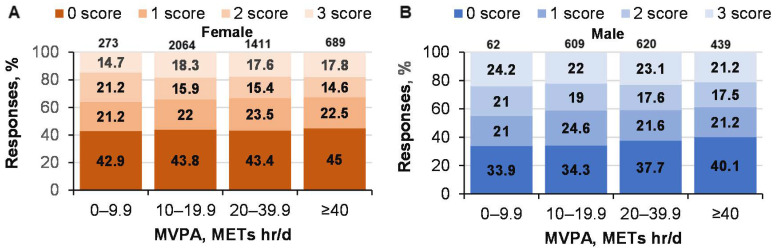
Solving CRT tasks in men and women of different ages with MVPA METs. ((**A**)—women CRT responses % and MVPA; (**B**)—men CRT responses % and MVPA).

The effect of solving CRT problems on MPA was not significant (*p* = 0.15; ${ŋ}_{P}^{2}$ = 0.001; OP = 0.46); although the interaction of logic and gender on MPA was weak, but significant (*p* = 0.031; ${ŋ}_{P}^{2}$ = 0.001; OP = 0.71) (Figure 3). However, MPA depended on age (*p* < 0.001; $\;{ŋ}_{P}^{2}$ = 0.031; OP = 1), but not on gender (*p* = 0.56; $\;{ŋ}_{P}^{2}$ < 0.001; OP = 0.096). There was a weak, but statistically significant, relationship between solving CRT tasks and VPA (*p* = 0.044; ${ŋ}_{P}^{2}$ = 0.001; OP = 0.66; age, *p* < 0.001; ${ŋ}_{P}^{2}$ = 0.013; OP = 1; gender, *p* < 0.001; $\;{ŋ}_{P}^{2}$ = 0.051; OP = 1; interaction not significant) (Figure 3). The influence of MVPA on solving CRT tasks was not significant (female: χ^2^ = 9.1; *p* = 0.43; male, χ^2^ = value 5.6; *p* = 0.77; Figure 4). There was no significant correlation between the effectiveness of solving CRT tasks and MVPA (r = −0.017; *p* > 0.05).

### 3.4. Relationship between Sports-Specific Activities and CRT Results

**Figure 5:**
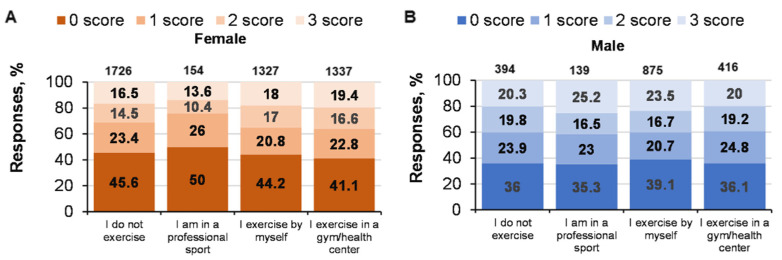
Solving CRT tasks and specific sporting activities. At the top of the column is the number of participants. ((**A**)—women CRT responses % and specific sporting activities; (**B**)—men CRT responses % and specific sporting activities).

Professional sportswomen solved all three CRT tasks less often (*p* < 0.05) compared with the other groups (Figure 5). Thus, women’s effectiveness in solving CRT tasks is linked with sport and the specificity of sporting activity (χ^2^ = 19.1; *p* = 0.01), but this relationship was not significant for men (χ^2^ = 8.7; *p* = 0.46). Interestingly, one fourth of professional sportsmen (the maximum from all the groups according to the specificity of sport) solved the tasks correctly, but just a little more than one third (the minimum according to the other specificities of sporting activity) did not solve at least one CRT task correctly.

**Figure 6:**
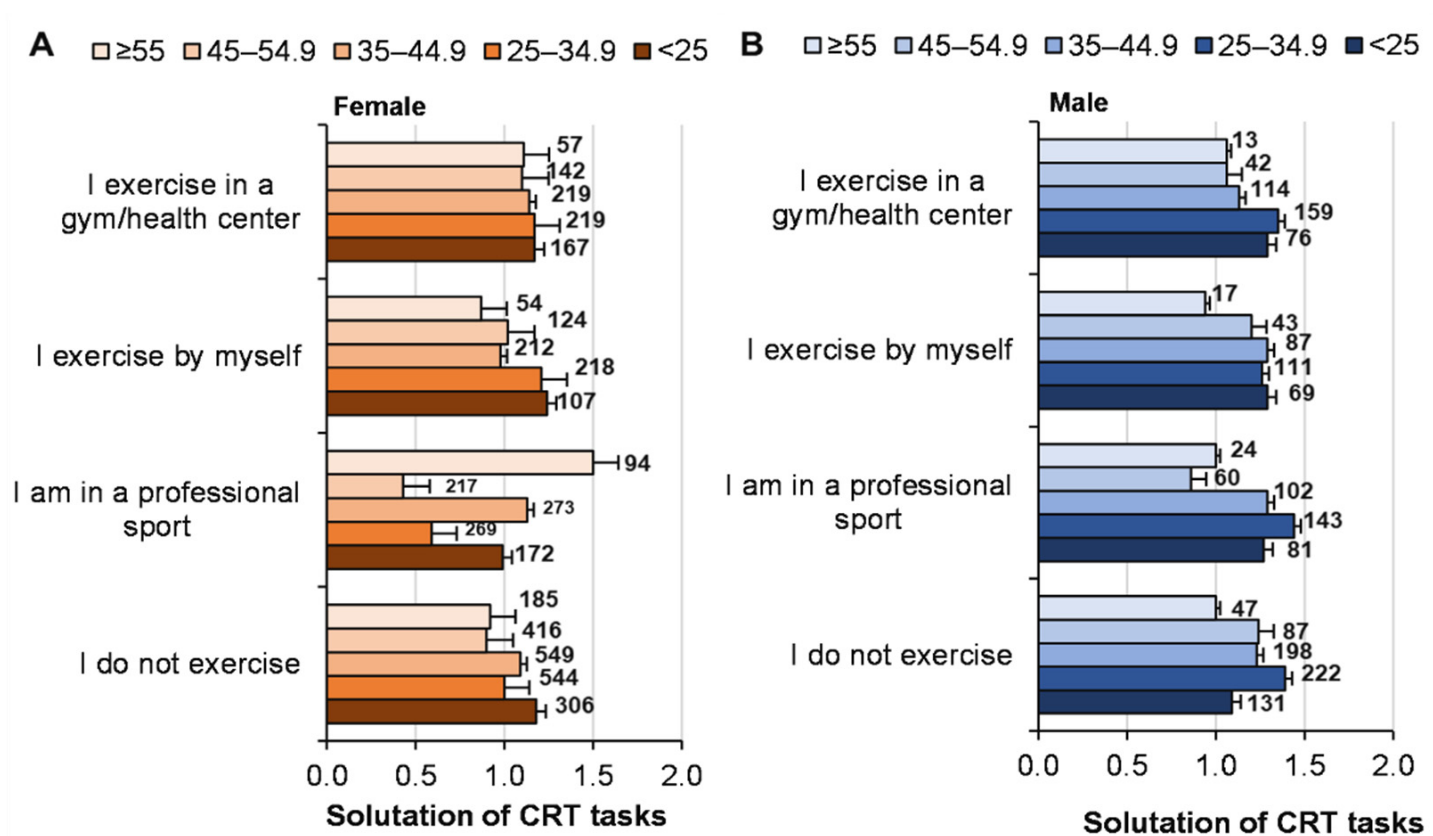
Solving CRT tasks and specific sporting activities depending on age and gender. At the top of the column is the number of participants. ((**A**)—women solutions of CRT and specific sporting activities depending on age; (**B**)—men solutions of CRT and specific sporting activities depending on age).

There was no statistically significant relationship between solving logical CRT tasks and specific sporting activities (*p* = 0.71; ${ŋ}_{P}^{2}$ < 0.001; OP = 0.14; age: *p* = 0.205; ${ŋ}_{P}^{2}$ = 0.001; OP = 0.49; gender: *p* = 0.052; ${ŋ}_{P}^{2}$ = 0.001; OP = 0.49; factor interaction, n.s.) (Figure 6).

**Figure 7:**
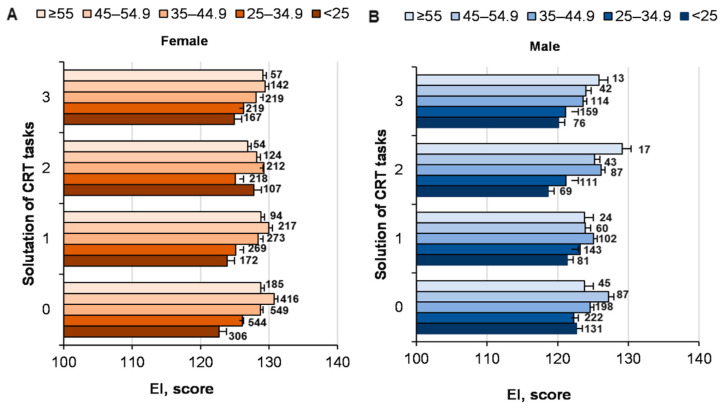
The EI of men and women of different ages in solving CRT tasks. At the top of the column is the number of participants. ((**A**)—women of different ages EI score and solutions of CRT; (**B**)—men of different ages EI score and solutions of CRT).

The influence of CRT tasks problem solving on EI was not significant (*p* = 0.83; ${ŋ}_{P}^{2}$ < 0.001; OP = 0.107), but the EI of women was higher (*p* < 0.001; ${ŋ}_{P}^{2}$ = 0.007; OP = 1) (Figure 7). With an increase in age from 18 to 64 years, the EI of men and women increased equally (*p* < 0.001; ${ŋ}_{P}^{2}$ = 0.01; OP = 1). The interaction of factors (problem solving, gender, age) was not significant (*p* > 0.05). We determined a statistically significant inverse correlation (Pearson’s coefficient) between the effectiveness of solving CRT tasks and EI (r = −0.049; *p* < 0.01).

### 3.5. Effectiveness of Solving CRT Tasks with Sociodemographic Factors and Healthy Lifestyle Factors

There were no statistically significant relationships between the effectiveness of solving CRT tasks and healthy lifestyle components, such as overeating, eating breakfast, alcohol consumption, and smoking (Table 1). Moreover, there were no statistically significant relationships between the effectiveness of solving CRT tasks and health, impulsivity, and sleeping patterns. However, the higher the stress level, the more women were depressed, and the worse they solved tasks in an logical way. However, women who lived in the countryside solved CRT tasks worse, while men who lived in a cottage or women who lived in a shared apartment solved them in the most effective way. Conversely, women who lived in a house solved tasks in the worst way. It is surprising that the effectiveness of solving CRT tasks did not depend on educational level.

We determined a statistically significant inverse correlation (Pearson’s coefficient) between the effectiveness of solving CRT tasks and stress (r = −0.048; *p* < 0.01).

**Figure 8:**
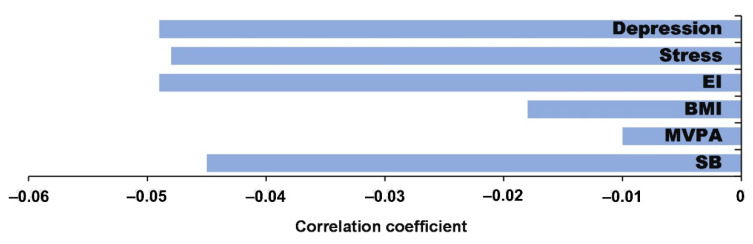
Correlation coefficient between CRT (LT) and depression, stress, EI, BMI, MVPA, and SB.

It is seen in Figure 8 that LT has the strongest inverse correlation with depression, stress, EI, and SB.

## 4. Discussion

To our knowledge, this is the first large-scale study to investigate the effectiveness of solving CRT tasks by men and women at different ages (18–74 years: i.e., logical vs. intuitive thinking) on the one hand and PA components (SB, MPA, VPA, MVPA), specific sporting activities, BMI, assessment of subjective health, sleep duration, EI, stress, depression, impulsivity, sociodemographic components, and healthy lifestyle components on the other hand. We did not find any significant relationships between the BMI of men and women at different ages and their effectiveness in solving tasks, except for BMI > 35 kg/m^2^, where the effectiveness decreased in both men and women (the tasks were more often solved by intuitive thinking). We did not find any significant relationships between PA level (MPA, VPA, and MVPA METs), sleep duration, impulsivity, healthy lifestyle components (nutrition, smoking, and alcohol consumption habits) in the assessment of health and the effectiveness of solving CRT tasks. However, the correlation was inverse and statistically significant for SB and effectiveness of solving CRT tasks (people with SB intuitively solved tasks more often). Moreover, there was an inverse correlation between EI, stress, and LT. On investigating the differences in solving CRT tasks between sedentary individuals, professional sportspersons, amateur sportspersons, and gym-goers, professional sportswomen solved CRT tasks in the worst way.

Our findings are consistent with [57], i.e., normal cognitive aging is characterized by nearly linear declines from early adulthood in speed and accelerating declines in memory and reasoning. The effectiveness of solving CRT tasks clearly decreases with age: thus, aging men and women switched from logical thinking to intuitive thinking.

We found that 36.7% of 18–25-year-old men (20.3% solved all three tasks) and 40.7% of women (16.5% solved all three tasks) did not solve at least one CRT task. This was similar to Frederick (2005) [44], because about 33% of the subjects did not solve the same CRT tasks (17% solved all tasks). Our data for solving CRT tasks were consistent with Frederick’s (2005) findings, i.e., men solved CRT tasks better than women did. Our findings supplement Fredrick’s (2005) finding that solving CRT tasks decreased in aging men and women (there was no difference in the effectiveness of solving CRT tasks between genders aged >55 years) [44].

Some cross-sectional studies suggest that PA improves sleep quality, which could be a mechanism by which PA improves cognitive abilities [58]. However, we did not find significant associations between sleep duration and quality of CRT task solving.

Obesity has also been consistently associated with deficits in cognitive abilities and brain health [24]. Hou (2019) showed that obesity was associated with a lower risk of cognitive impairment in Chinese subjects aged ≥60 years, whereas abdominal obesity was associated with an increased risk of cognitive impairment, independent of conventional sociodemographic, lifestyle, and health-related comorbid factors [59]. Cook et al. (2017) found that obese women had normal but significantly lower attentional performance and were more impulsive than normal participants [60]. Specifically, in the context of executive functions (e.g., inhibitory control, set-shifting, working memory, decision-making), higher BMI is associated with poorer task performance [61]. Morys (2021) found that BMI was positively related to higher plasma C-reactive protein, dyslipidemia, hypertension, and diabetes [29]. Hypertension and diabetes, in turn, were associated with cerebrovascular disease. Finally, cerebrovascular disease was associated with cognitive deficits and lower cortex thickness and volume and higher subcortical volume. Our results confirm this finding, as only subjects with a BMI > 35 kg/m^2^ solved the tasks intuitively more often than logically.

Studies in humans have shown that PA is associated with an increase in peripheral brain-derived neurotrophic factor (BDNF) [22]. Higher concentrations of peripheral BDNF following participation in a sports intervention mediated improvements in executive function [62] and memory [63]. Exercise-induced increases in serum BDNF levels also correlated with greater hippocampal volume [20] following exercise, supporting the hypothesis that exercise promotes brain health through modulation of BDNF signaling pathways. Despite the attractive hypothesis that PA should improve cognitive function, our research did not show a significant association between PA intensity (MPA, VPA, or MVPA) and intuitive or logical thinking. However, we found an inverse correlation between SB and the correct solution of a task. In other words, people who sat for longer more often solved tasks intuitively. However, this partially contradicts [64], who showed that cognitive function in healthy women (aged 18–35 years) did not depend on PA or sitting time. Thus, we still need clearer proof for whether PA improves reasoning and decision-making during daily life. Ref. [43] observed that boosting intelligence via multimodal intervention is effective even in young, healthy adults, and is a promising avenue to improve reasoning and decision-making in daily life. Other studies have shown that EI is related to human health [65,66], PA [4], and rapid decision-making [67]. However, there were no significant relationships between LT and components of a healthy lifestyle (eating habits, sleep quality, smoking, and alcohol consumption). We found an inverse correlation between LT and EI. Thus, there are two different systems of thinking [68].

Frederick (2005) found that people who solved CRT tasks in the best way did not choose a rapid and small reward, but rather chose a big reward later [44]. An individual’s choice of PA is also affected by a number of interrelated determinants, such as demographic characteristics, health and health behavior, and psychological, social, and environmental determinants related to the intervention [69,70]. Thus, it is quite difficult to explain why professional sportswomen analytically solved tasks especially poorly. It should be investigated whether this result depended on genetic factors or because they had less time to mentally train because of their high physical loads.

## 5. Limitations and Directions for Future Research

The main limitation of our research was the PA questionnaire because it might slightly overestimate PA. Danish studies have shown that the scale we also used overestimated the time spent on light, moderate, and vigorous intensity PA and underestimated the time spent in SB [12]. In addition, it is difficult to compare PA data given the variety of methodologies for determining PA [2,71,72]. Moreover, we only considered two aspects of cognitive thinking, logical and intuitive thinking, and did not investigate any executive functions, which did not enable our data to be more widely compared with other studies. Of course, there are many more LT-affecting factors and they are often “intertwined”, so it is difficult to accentuate which ones are the main ones, what the reason is, and what the consequence is.

## 6. Practical Implications of the Study

We think the biggest practical benefit of our study is that the strongest determinants, which reduce LT, are SB, obesity, depression, and stress. Thus, by eliminating them (for example, by increasing PA), LT can be improved. Our previous studies have shown clearly that PA improves EI especially [9]. It coincides with the previous meta-analyses which confirm that EI improves mental health and reduces stress [65,73].

## 7. Conclusions

We found that neither intuitive thinking nor LT is associated with sleep, MPA, VPA, MVPA, impulsivity, health, and healthy lifestyle components, but LT depends on age (it decreases), gender (higher in men than in women), BMI (decreases in both genders over the second degree of obesity), depression (the more severe the depression in women, the worse the LT), SB (people who sat longer had worse solutions for the tasks), and on professional sportswomen (LT was worse in professional sportswomen than among other women or in women who attend gyms). Finally, we determined inverse correlations between LT, EI, and stress. Thus, although the relations between logical thinking and wellbeing, PA, and physical health are complicated, there are no doubts that PA creates favorable conditions for the improvement of mental health through the improvement of health and wellbeing.

## Figures and Tables

**Table 1:** Relationships between the effectiveness of solving CRT tasks and sociodemographic and healthy lifestyle factors.

Figure	Chi-Square		*p* Value	
	Female	Male	Female	Male
Education	11.7	17.6	0.69	0.28
Residential place (city vs. country)	48.1	11.3	<0.001	0.25
Married vs. single	4.4	12.9	0.88	0.16
Type of work	14.2	7.7	0.077	0.56
Residential place (house)	38.1	27.8	<0.001	<0.001
Breakfast	2.1	5.8	0.9	0.43
Overeating	11.4	7.3	0.075	0.28
Smoking	15	11.2	0.089	0.026
Alcohol	13.2	17.7	0.77	0.47
Health	5.2	3.9	0.83	0.91
Stress	17.2	13.9	0.009	0.03
Impulsivity	8.7	9.4	0.45	0.41
Depression	19.3	12.6	0.023	0.175
Sleep	15.8	8.8	0.19	0.71

## Data Availability

The data presented in this study are available on request from the corresponding author.
